# [Retracted] ANRIL is associated with the survival rate of patients with colorectal cancer, and affects cell migration and invasion  *in* *vitro*

**DOI:** 10.3892/mmr.2024.13354

**Published:** 2024-10-07

**Authors:** Yi Sun, Zhao-Peng Zheng, Hang Li, Han-Qun Zhang, Fa-Qiang Ma

Mol Med Rep 14: 1714–1720, 2016; DOI: 10.3892/mmr.2016.5409

Following the publication of this paper, it was drawn to the Editors’ attention by a concerned reader that the Transwell migration and invasion assay data shown in Fig. 4A and B on p. 1418 were strikingly similar to data appearing in different form in other articles written by different authors at different research institutes that had already been published elsewhere prior to the submission of this paper to *Molecular Medicine Reports*. In view of the fact that the abovementioned data had already apparently been published previously, the Editor of *Molecular Medicine Reports* has decided that this paper should be retracted from the Journal. The authors were asked for an explanation to account for these concerns, but the Editorial Office did not receive a reply. The Editor apologizes to the readership for any inconvenience caused.

